# Transcriptomic and metabolomic analyses reveal how girdling promotes leaf color expression in *Acer rubrum* L

**DOI:** 10.1186/s12870-022-03776-6

**Published:** 2022-10-24

**Authors:** Yan Yangyang, Liu Qin, Yan Kun, Wang Xiaoyi, Xu Pei

**Affiliations:** 1 Institude of mountain hazards and environment, Chinese Academy of Sciences, 610041 Chengdu, China; 2 Key Station of Ecological Environment Monitoring in Typical Area of Wanzhou, Wanzhou, 404100 China; 3 College of Water Conservancy and Hydropower Engineering, Sichuan Agricultural University, 625014 Ya’an, China

**Keywords:** *Acer rubrum* L., Girdling, Leaf coloration, Physiological, Anthocyanin, Chlorophyll, Transcriptome, Metabolomics

## Abstract

**Background:**

*Acer rubrum* L. (red maple) is a popular tree with attractive colored leaves, strong physiological adaptability, and a high ornamental value. Changes in leaf color can be an adaptive response to changes in environmental factors, and also a stress response to external disturbances. In this study, we evaluated the effect of girdling on the color expression of *A. rubrum* leaves. We studied the phenotypic characteristics, physiological and biochemical characteristics, and the transcriptomic and metabolomic profiles of leaves on girdled and non-girdled branches of *A. rubrum*.

**Results:**

Phenotypic studies showed that girdling resulted in earlier formation of red leaves, and a more intense red color in the leaves. Compared with the control branches, the girdled branches produced leaves with significantly different color parameters a*. Physiological and biochemical studies showed that girdling of branches resulted in uneven accumulation of chlorophyll, carotenoids, anthocyanins, and other pigments in leaves above the band. In the transcriptomic and metabolomic analyses, 28,432 unigenes including 1095 up-regulated genes and 708 down-regulated genes were identified, and the differentially expressed genes were mapped to various KEGG (kyoto encyclopedia of genes and genomes) pathways. Six genes encoding key transcription factors related to anthocyanin metabolism were among differentially expressed genes between leaves on girdled and non-girdled branches.

**Conclusions:**

Girdling significantly affected the growth and photosynthesis of red maple, and affected the metabolic pathways, biosynthesis of secondary metabolites, and carbon metabolisms in the leaves. This resulted in pigment accumulation in the leaves above the girdling site, leading to marked red color expression in those leaves. A transcriptome analysis revealed six genes encoding anthocyanin-related transcription factors that were up-regulated in the leaves above the girdling site. These transcription factors are known to be involved in the regulation of phenylpropanoid biosynthesis, anthocyanin biosynthesis, and flavonoid biosynthesis. These results suggest that leaf reddening is a complex environmental adaptation strategy to maintain normal metabolism in response to environmental changes. Overall, the results of these comprehensive phenotype, physiological, biochemical, transcriptomic, and metabolomic analyses provide a deeper and more reliable understanding of the coevolution of red maple leaves in response to environmental changes.

## Highlights

1. Comparisons between leaves on girdled and non-girdled branches of red maple showed that leaf color change is a coevolutionary phenomenon of maple in response to abiotic stress.

2. C108619.graph_c0 of Pkinase family regulates the color change of *A. rubrum* leaf by participating in the synthesis of anthocyanins, which has the high homology with c45897.graph_c0 and* OsEDR1* of *Lagerstroemia indica*.

## Background

*Acer rubrum* L. (red maple) is native to the eastern part of North America and is mainly distributed in Canada and the United States. It has strong physiological adaptability and a high ornamental value. Consequently, it is a very popular landscape plant [1]. It was introduced into China in the 21st century [2], first to botanical gardens and arboretums, then into research and germplasm conservation facilities at scientific research institutes and universities, and finally into horticultural companies for sale to the general public. Breeders and researchers have aimed to select and breed garden cultivars with excellent performance under local environmental conditions, and to promote their cultivation in Chinese gardens [3, 4]. However, in Chongqing and other southern regions, the coloration time and quality of red maple are not ideal because of certain environmental conditions such as low light levels and temperatures in autumn.

Many studies have shown that environmental conditions such as light, temperature, and soil conditions strongly affect leaf color change in colored-leaf plants. This is because such conditions affect the synthesis and accumulation of pigments in plants [5]. In nature, leaf color change is affected by the combined actions of multiple factors, but changes in any one factor can also cause the leaf color to change [6]. In recent years, researchers have studied the regulation of plant leaf color from the perspectives of gene regulation, light intensity regulation, and the effects of exogenous nutrients applied by spraying [7–9]. The genes involved in the anthocyanin synthesis pathway can be divided into two types [10, 11]: structural genes, which are found in all plants and directly encode anthocyanin biosynthetic enzymes; and regulatory genes, which encode proteins that regulate the activity structural genes, and therefore, the accumulation of pigments, in space and time. As one of the most important environmental factors involved in anthocyanin synthesis, light regulates the synthesis and quantity of anthocyanins via photoreceptors and light-signal transduction factors [12]. In general, strong light induces the expression of anthocyanin structural and regulatory genes, leading to anthocyanin accumulation, while darkness or weak light inhibits or down-regulates the expression of those genes, thus inhibiting anthocyanin synthesis [13].

Anthocyanin synthesis is also related to the sugar content in plants. Previous studies have shown that exogenous sucrose treatment can significantly increase the sugar content in plant leaves, promote anthocyanin accumulation, and affect the appearance of leaf color [14]. It was reported that sucrose could promote anthocyanin synthesis in suspension cell cultures [15]. Spraying leaves of safflower maple with 0.2 mol/L sucrose solution did not markedly affect the chlorophyll content, but significantly increased the anthocyanin content, confirming that exogenous sugar could promote leaf color development [16]. In another study, ring-cutting not only promoted the accumulation of sucrose, glucose, and fructose in leaves of maple, but also increased the anthocyanin content in red leaves from 50.4 to 66.7%, and that in original yellow leaves from 11.7 to 54.2%, which significantly enhanced leaf color expression [14]. Girdling has been used in plant research for a long time, but it has mainly been used to promote fruiting performance in fruit trees. Many studies have focused on the changes in nutrient transport and distribution after ring-cutting. However, few reports have focused on the use of ring-cutting to regulate leaf color [17, 18].

*A. rubrum* is a non-model plant, and consequently, its genome has not been fully sequenced and bioinformatics information is very scarce. With the increasing maturity of sequencing technology, high-throughput sequencing technology can detect transcripts in all species without the requirement for genomic information. Thus, it can be used to study the leaf coloration mechanisms of *A. rubrum*. RNA-Seq sequencing technology can yield a large amount of genetic information, which can shed light on the biochemical and regulatory aspects of plant secondary metabolism. Thus, this technology can provide new opportunities to study changes in leaf color in colored-leaved plants [19, 20].

In this study on *A. rubrum*, the leaf color parameters and related physiological indexes were compared between girdled and non-girdled branches to reveal the physiological mechanism of the red coloration of the leaves in autumn. At the same time, a high-throughput sequencing platform (Illumina HiSeq) was used to analyze the transcriptome of leaves from girdled and non-girdled branches. Differentially expressed genes were identified and mapped to KEGG pathways. These analyses provide new information about the molecular mechanism and theoretical basis of leaf color variation in red maple.

## Results

### Chlorophyll and carotenoid concentrations in leaves collected from three different positions

**Fig. 1:**
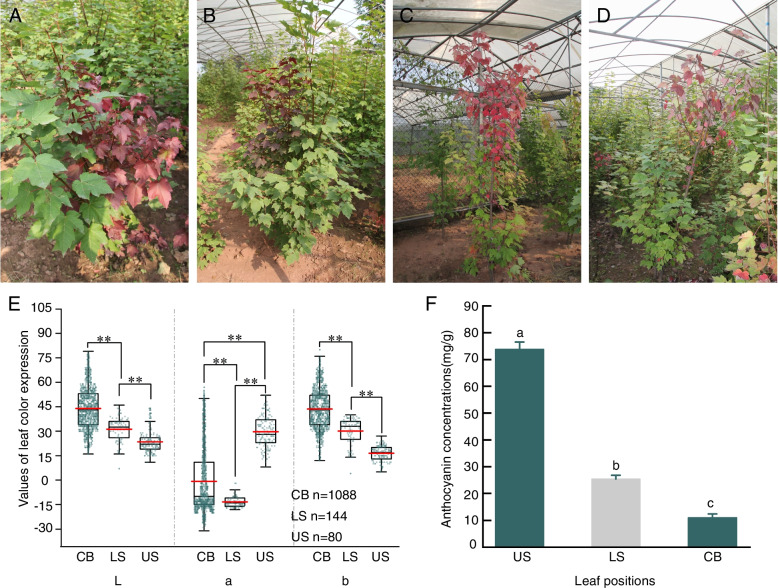
**A**, **B**, **C**, **D** show the repeated experiments, which the change of foliar color between the treated bunches and control branches. Based on the selection, a branch from the tree was ligated tightly with plastic strip in middle part of the branch and tagged on each tree. **E** shows the variation of parameters L, a, b. **F** shows the difference in the leaf anthocyanin between the treated bunches and control branches. (US represents the upper stem; LS represents the lower stem; CB represents the control branch)

Leaves were collected from non-girdled branches (CK) and from above and below the girdling site on girdled branches. Girdling affected color formation in leaves of *A. rubrum.* Leaves from above the girdling site turned uniformly red, while leaves below the girdling site and on control branches remained green (Fig. 1A, B, C, D). Statistical analyses (ANOVA and Tukey–Kramer test results) of L a* b* values of leaves on treated and control branches revealed significant differences, especially in parameters a* (Fig. 1E). Although leaves from CK and those below the girdling site had the same color and carotenoid concentrations, the leaves from below the girdling site had higher chlorophyll a, chlorophyll b, and anthocyanin contents, compared with CK leaves (Table 1), and the differences were significant for chlorophyll b and chlorophyll (a + b) (*P* = 0.02). The leaves above the girdling site were bright red, and contained much higher contents of anthocyanins and significantly lower contents of chlorophyll a, chlorophyll b, and carotenoids, compared with leaves below the girdling site and those from CK (*P* < 0.05) (Table 1; Fig. 1F).

**Table 1:** Chlorophyll a, b, a + b and carotenoid, anthocyanin concentrations of leaves from upper, lower and control stems (means ± SE). Different letters (a, b and c) behind the data indicate significant (*P* < 0.05) differences between leaf positions

Leaf position	Chlorophyll a (mg/g)	Chlorophyll b (mg/g)	chlorophyll (a + b) (mg/g)	Carotenoid (mg/g)
Upper stem	198.64 ± 27.85b	140.57 ± 30.26c	339.21 ± 32.12c	69.50 ± 20.61b
Lower stem	1889.76 ± 437.50a	624.14 ± 72.54a	2077.90 ± 108.70a	276.85 ± 123.42a
Control branch	1305.46 ± 15.74a	432.54 ± 12.84b	1738.01 ± 19.24b	279.65 ± 8.84a

The pigment ratios were not significantly different between leaves from the below the girdling site and leaves from CK (*P* > 0.05) (Fig. 1A, B, C, D). Nevertheless, the ratio of chlorophyll/carotenoids to anthocyanins was lower in the leaves below the girdling site than in CK leaves (*P* < 0.05). Compared with the CK leaves and leaves from below the girdling site, the red leaves above the girdling site had lower ratios of all the pigment parameters, but the differences in chlorophyll a:b and chlorophyll: carotenoid ratios were not significant. The highest ratio of anthocyanins to chlorophyll was in the leaves above the girdling site (Table 2).

**Table 2:** Ratios between pigments in different leaf positions (means ± SE). Different letters (a, b and c) behind the data indicate significant (*P* < 0.05) differences between leaf positions

Leaf position	Chlorophyll a/b	Chlorophyll/carotenoid	Chlorophyll/anthocyanin	Carotenoid/Anthocyanin
Upper stem	1.57 ± 0.66a	6.62 ± 2.97a	4.64 ± 0.58c	0.94 ± 0.27b
Lower stem	3.08 ± 1.23a	6.18 ± 1.70a	81.91 ± 1.82b	15.65 ± 4.48a
Control branch	3.02 ± 0.17a	6.23 ± 0.18a	162.12 ± 18.92a	26.25 ± 3.71a

### Soluble sugars and flavonoid concentrations in leaves from three positions

**Fig. 2:**
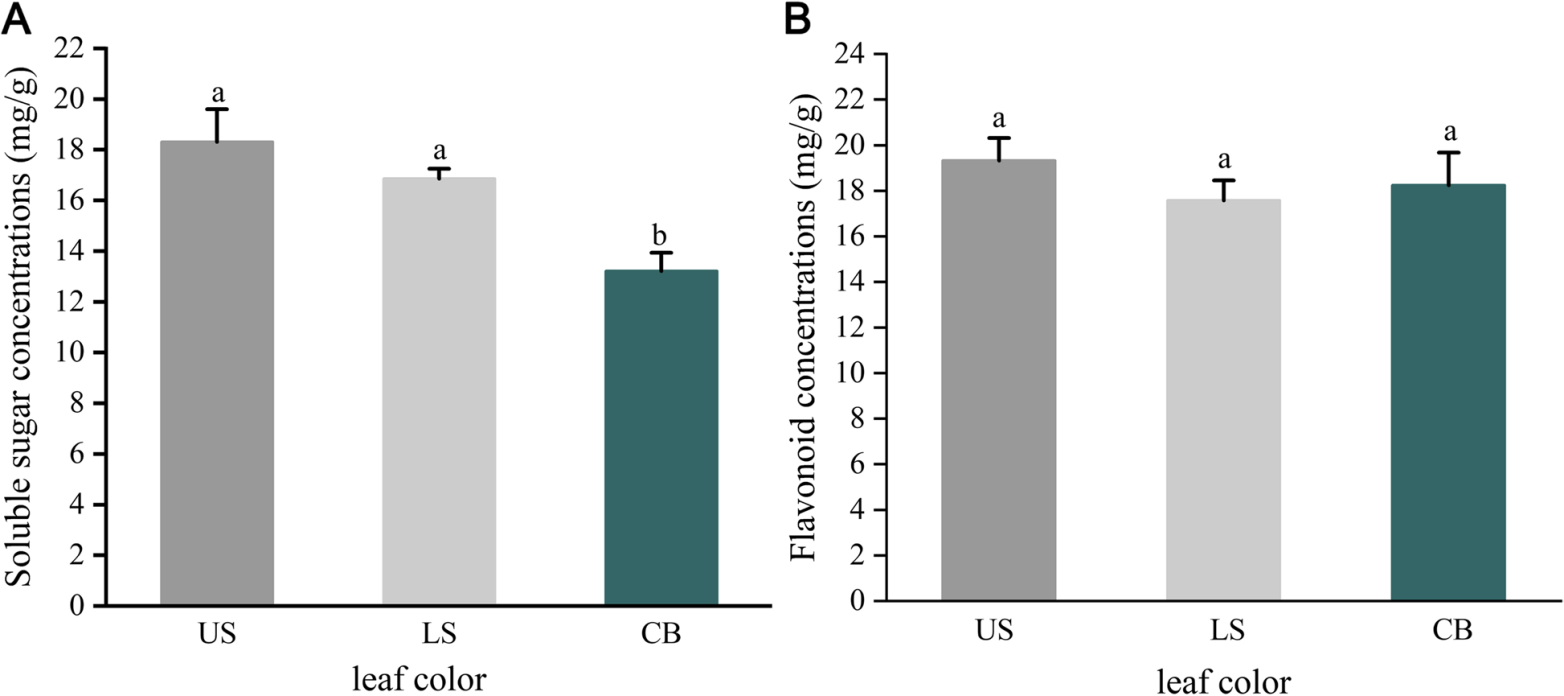
Soluble sugar and flavonoid concentrations of leaves collected from upper stem, lower stem and control branch. Standard error bars represent ± 1 SE. Different letters (a and b) behind the data indicate significant (*P* < 0.05) differences between leaf positions. (US represents the upper stem; LS represents the lower stem; CB represents the control branch)

Disruption of phloem transport by girdling resulted in measurable increases in soluble sugars concentrations in the leaves (Fig. 2A). Compared with leaves from CK, those from above and below the girdling site on girdled branches showed increased soluble sugars contents (Fig. 2A). Although the soluble sugars content was slightly lower in the leaves below the girdling site than in the leaves above the girdling site, the levels were comparable. The flavonoid concentrations did not differ significantly among the CK leaves, the leaves from above the girdling site, and the leaves from below the girdling site (Fig. 2B).

### Transcript profiles of leaves from girdled and non-girdled branches

**Fig. 3:**
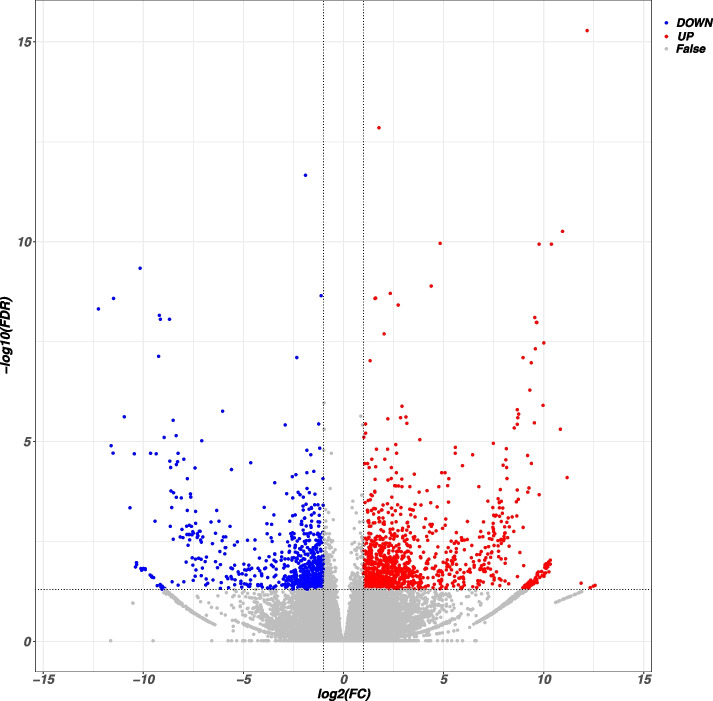
Statistical analysis of all differentially expressed genes (DEGs) Volcano plot of DEGs

The RNA extracted from leaves of six CK samples (CK) and six girdled-branch samples (TT) was sequenced, yielding RNA a total of 7.31 Gb of clean data. There were 1803 differentially expressed genes (DEGs) between CK and the girdled group, including 1095 up-regulated genes and 708 down-regulated genes (Fig. 3).

**Fig. 4:**
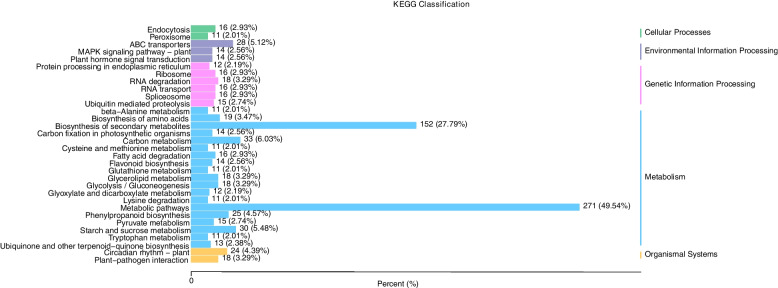
KEGG pathway annotation of *Acer rubrum* L. transcripts. The vertical axis shows the annotations of the KEGG metabolic pathways. The horizontal axis represents the gene numbers annotated in each pathway

**Fig. 5:**
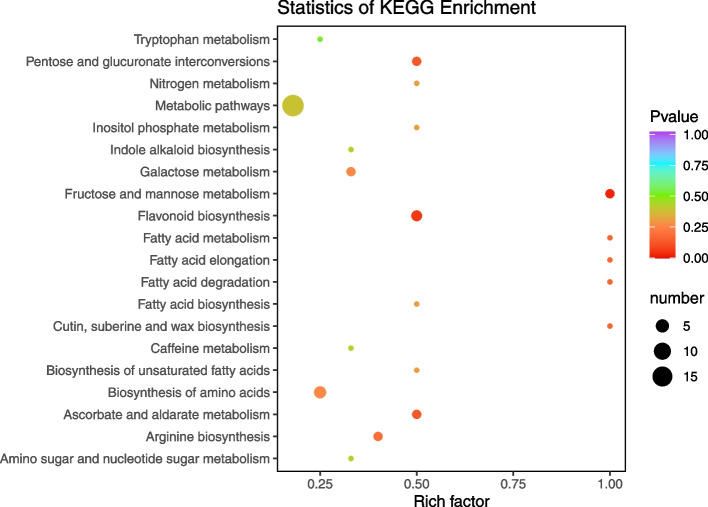
Statistical analysis of KEGG pathway enrichment of up-regulated DEGs. Each circle represents a KEGG pathway, the ordinate represents the pathway name, and the abscissa is the enrichment factor. The larger the enrichment factor is, the greater the degree of enrichment is. The circle color represents q-value, the smaller the q-value is, the more reliable the enrichment significance is. The size of circle indicates the number of genes enriched in the pathway, and the larger the circle, the more abundant the genes is

A total of 28,432 unigenes were mapped to KEGG pathways. The three pathways with the largest number of unigenes were metabolic pathways (ko01100, 12,563 unigenes, 49.54%), biosynthesis of secondary metabolites (ko01110, 5864 unigenes, 27.79%), and carbon metabolism (ko01200, 1695 unigenes, 6.03%) (Figs. 4 and 5).

**Fig. 6:**
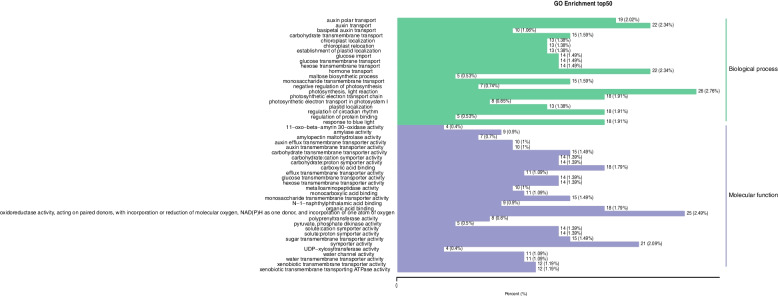
Go enrichment histogram of differential genes. The horizontal axis shows the ratio of the genes annotated to the total number of genes annotated. The ordinate represents the name of the Go entry. The label to the right of the figure represents the category to which the Go entry belongs

In a gene ontology (GO) functional enrichment analysis, the top five significantly enriched functional categories were photosynthesis, light reaction (2.76%), oxidoreductase activity (acting on paired donors with incorporation or reduction of molecular oxygen, NAD(P)H as one donor, and incorporation of one atom of oxygen) (2.49%), hormone transport (2.34%), auxin transport (2.34%), and symporter activity (2.09%) (Fig. 6).

**Fig. 7:**
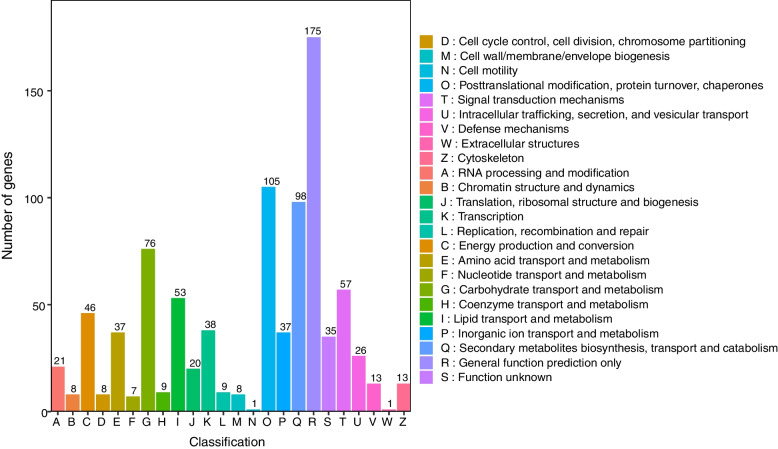
KOG classification of *Acer rubrum* L. transcripts. The capital letters on the horizontal axis indicate the KOG categories,which are explained below the histogram, and those on the vertical axis indicate the number of genes

There were 28,536 DEGs between CK and TT, including 5556 up-regulated and 3054 down-regulated unigenes. Flavonoids are important secondary metabolites, and many pigments are synthesized via the flavonoid biosynthesis pathway. In the comparison of CK vs. TT, several DEGs were enriched in the flavonoid biosynthetic pathway (14 up-regulated and two down-regulated unigenes). In the eukaryotic orthologous groups (KOG) analysis, 69,181 unigenes (55.15%) were grouped into 25 KOG classifications. Group R (general function prediction only) was the most highly represented category. Large proportions of unigenes were in Group T (signal transduction mechanisms) and Group O (post-translational modification, protein turnover, chaperones) (Fig. 7).

### Metabolomic characteristics of leaves on girdled branches

**Fig. 8:**
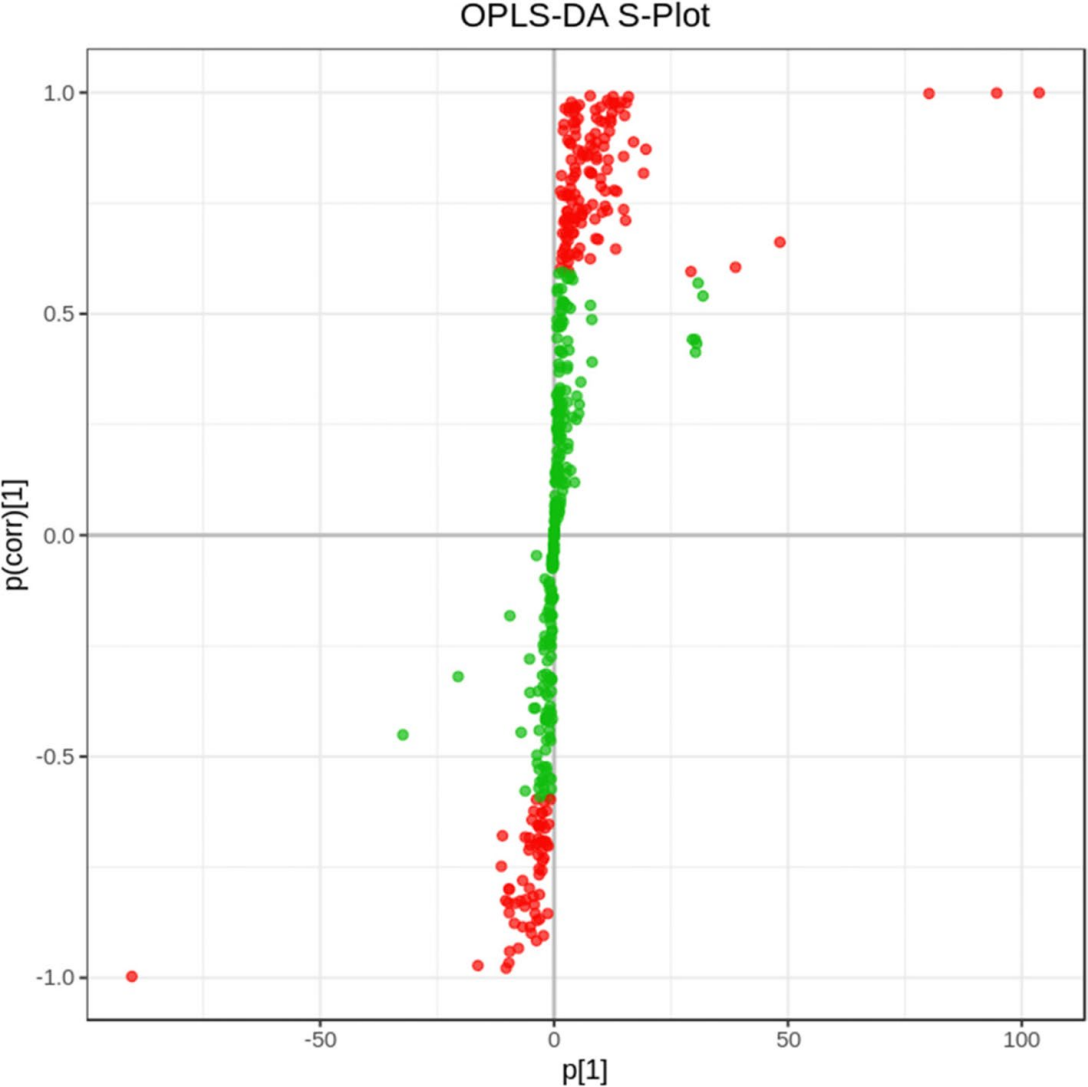
OPLS-DA S-plot (The abscissa represents the covariance between principal components and metabolites, and the ordinate represents the correlation coefficient between principal components and metabolites. The metabolites closer to the upper right corner and lower left corner in the figure indicate more significant differences. Red dots indicate that the VIP of these metabolites is ≥ 1, while green dots indicate that the VIP of these metabolites is < 1.)

Untargeted metabolomics analysis of *A. rubrum* leaves from CK and TT was conducted using liquid chromatography- mass spectrometry (LC-MS). A total of 442 compounds were identified, including saccharides, flavonoids, carbohydrates, and amino acids and their derivatives. The results of the orthogonal partial least squares discriminant analysis (OPLS-DA) in anion and cation mode revealed 79 differentially accumulated metabolites between CK and TT, of which 63 were up-regulated and 16 were down-regulated (filtering criteria: *P* < 0.05 and variable importance in projection value (VIP) > 1) (Fig. 8). Multivariate analysis of the VIP values from the OPLS-DA model further identified differentially accumulated metabolites between CK and TT. This method, combined with the Fold Change method, identified 10 metabolites with significant differences and VIP ≥ 1 between CK and TT, of which eight were up-regulated and two were down-regulated.

### Effects of girdling on transcriptional factors related to anthocyanin metabolism

Six genes encoding transcription factors related to anthocyanin metabolism were among the DEGs between CK and TT. They encoded members of kinase Pkinase (c108619.graph_c0), UDPGT(c117950.graph_c0), Metallophos (c115191.graph_c0), amp-binding (c109312.graph_c0), UDP-GT(C122287.graph_c0), and DIOX_N (C105756.graph_c0) families that are known to regulate leaf color in *A. rubrum* via modulation of phenylpropanoid biosynthesis, anthocyanin biosynthesis, and flavonoid biosynthesis. To explore the transcriptional patterns of these six transcription factors during leaf coloration in red maple, we analyzed their transcript levels in girdled and CK branches. The results showed that C105756.graph_c0, C109312.graph_c0, and C117950.graph_c0 were most highly expressed in red leaves, and C122287.graph_c0, c115191.graph_c0, and c108619.graph_c0 were most highly expressed in leaves and flowers. These findings indicated that these six transcription factors play a key role in leaf color change in red maple (Table 3).

**Table 3:** The DEGs associated with anthocyanin and GO annotation

Gene ID	Gene length/bp	Gene family	GO annotation	FC	*P* value
c108619.graph_c0	4952	Pkinase	Regulation of anthocyanin biosynthetic process	-1.5749	0.0016
c122287.graph_c0	5984	UDPGT	Anthocyanin-containing compound biosynthetic process	-1.4082	0.0060
c105756.graph_c0	2674	DIOX_N	Anthocyanin-containing compound biosynthetic process	-1.6534	0.0001
c109312.graph_c0	2804	AMP-binding	Anthocyanin-containing compound biosynthetic process	-1.9340	0.0001
c115191.graph_c0	1742	Metallophos	Regulation of anthocyanin metabolic process	3.0563	0.0004
c117950.graph_c0	3485	UDPGT	Anthocyanin 5-O-glucosyltransferase activity	-1.8209	0.0003

## Discussion

### Effect of girdling on leaf color expression

The color of upper leaves differed significantly between girdled and non-girdled branches. The color of upper leaves on girdled branches changed to red in mid-October and the branches defoliated in mid-November, while leaves on branches below the girdling site and on control branches turned red in mid-November and defoliated in mid-December. Stem girdling with a glass fiber shortened the duration of the red coloration of the leaves, but enhanced its uniformity and brilliancy. This may be because girdling interfered with photosynthesis and promoted pigment formation and accumulation. Consistent with this, our results show that girdling resulted in the accumulation of anthocyanins. Phloem disruption in branches of red maple caused the uneven accumulation of pigments including chlorophyll, carotenoids, and anthocyanins, in the leaves above the girdle. The anthocyanin contents were significantly higher in leaves above the girdling site than in those below the girdling site, consistent with patterns observed in other trees [21, 22]. Anthocyanins are water-soluble pigments that are responsible for the red, blue, and purple colors of various organs in diverse plants. In addition to conferring color, anthocyanins can function to alleviate photoinhibition and oxidative stress, and mitigate the effects of drought [23, 24]. This suggests that the accumulation of anthocyanins in girdled branches may be a protective response to external disturbances (e.g., light protection). Anthocyanins are stable and display a red color at lower pH, but become unstable and display blue or purple colors at higher pH [25]. Green leaves from control and lower branches had high chlorophyll a concentrations, suggesting that they were in the stage of accumulating carbohydrate reserves and needed chlorophyll for photosynthesis during autumn [26, 27].

Girdling not only affected anthocyanin contents, it also led to the decrease of chlorophyll b and total chlorophyll in the leaves above the girdling site (compared with CK), which would inhibited photosynthesis. Girdling also increased the soluble sugars content in leaves. There is a source–sink balance in plants. In trees, the starch content is highest in autumn and during the dormant season, decreases in spring during bud break, and is then replenished during summer and early autumn [28–30]. As observed in this study, girdling interfered with the transport of assimilates toward the roots through the phloem, leading to the accumulation of soluble sugars in branches above the girdling site, as observed previously [31]. In this study, girdle bands were applied to primary or secondary branches of 4-year-old *A. rubrum*. Leaves were harvested from control branches from top to bottom while the girdled branches were divided into two parts by the glass fiber. Leaves from the lower part were shaded by upper and control branches, and so they may have accumulated chlorophyll b as a response to shading, to capture more light for photosynthesis. Significantly high chlorophyll b and total chlorophyll concentrations in leaves on lower branch sites may improve light-interception efficiency [32]. As well as external shading, leaves may be subject to interior shading of mesophyll chloroplasts by anthocyanins. In addition, anthocyanin accumulation has a photosynthetic cost resulting from the competition between anthocyanins and chlorophylls to capture light [33]. As a result, compared with green leaves, red leaves have lower maximum CO_2_ assimilation rates, lower chlorophyll a/b ratios, and a smaller pool of xanthophyll cycle components [32, 34]. In this study, the chlorophyll a/b ratios were slightly lower in red leaves than in green leaves, but the difference was not significant (Table 2). The chlorophyll b concentration was lower in red leaves than in green leaves, suggesting that external shading had a stronger effect on leaf photosynthesis than did interior shading.

### Changes in leaf color may be indicative of coevolution of red maple and the environment

The color change in red maple leaves is affected by both genetic and environmental factors. Together, these factors regulate the change to deep red in autumn and under low temperature in winter, and the color change of new leaves from green to red in spring [35, 36]. The results of the present study show that girdling can also promote the red color formation in leaves. The contents of chlorophyll, carotenoids, and anthocyanins significantly differed among the leaves in different treatments, indicating that leaf color may be an indicator of plant adaptation to external disturbances and environmental changes. At present, the mainstream interpretation of the ecological significance of changes in leaf color is that it represents adaptation to biotic or abiotic stress, and has a photoprotective function [37]. It has been hypothesized that anthocyanins in red leaves block certain wavelengths of strong light and reduce damage caused by oxygen free radicals and superoxides produced via photosynthesis [38, 39]. In this study, girdling also led to premature leaf discoloration and significantly enhanced photosynthesis, suggesting a possible trade-off between leaf reddening and leaf mechanical protection. This study also supports the coevolutionary hypothesis of red leaves and other organisms.

### Effects of girdling on transcriptomic and metabolomic profiles of red maple leaves

Changes in leaf color can affect the ecological function of trees. To explore this topic in more detail, we analyzed the transcriptomic and metabolomic profiles of leaves from girdled and non-girdled branches. The results indicated that the leaf color change caused by girdling was mainly a result of changes in the carbon metabolic pathway in the leaves. The girdling disrupted the sieve tubes in the phloem, blocking the flow of assimilates, which caused carbohydrates to accumulate above the girdle band [40]. At the same time, assimilates accumulated in leaves. The accumulation of assimilates in tobacco leaves was shown to enhance carbon metabolism in tobacco plants, similar to the results of our study [41]. Photosynthetic pigments play a key role in the primary light reaction of photosynthesis, and changes in their concentrations are related to leaf physiological activity, plant adaptation to the environment, and responses to stresses [42]. On the one hand, the synthesis of photosynthetic pigments in plants is affected by stress. On the other hand, a feedback regulation mechanism operates to reduce the photosynthetic rate. In this study, compared with control branches, the girdled branches produced leaves with significantly lower contents of chlorophyll a, chlorophyll b, total chlorophyll and carotenoids. A decrease in photosynthetic pigment content reduces the photosynthetic rate of plants, and stress on the phloem can cause serious damage in the short term. Murakami et al. performed ring-cutting on maple trees with two leaf colors and found that mechanical damage not only promoted the accumulation of sucrose, glucose, and fructose in maple trees, but also increased the anthocyanin content in red leaves from 50.4 to 66.7%, and that in original yellow leaves from 11.7 to 54.2% [14]. Thus, girdling has a significant promoting effect on leaf color expression. The results of that study and our study suggest that leaf color is a morphological change representative of plant adaptation to the environment.

GO function enrichment results shows that differentially expressed genes associated with biological process is mainly enriched in metabolic processes, cellular processes. KEGG analysis showed that the differentially expressed genes were mainly enriched in metabolic pathways, biosynthesis of secondary metabolites, ribosomes, carbon metabolism, amino acid biosynthesis, glycolysis/gluconeogenesis, purine metabolism, flavonoid biosynthesis, glutathione metabolism, plant hormone signal transduction, and plant pathogen interaction. These metabolic pathways involved in energy, carbohydrate, amino acid and polysaccharide biosynthesis, metabolism, and some signal transduction process of life activity, further illustrates the obtained a lot of information through the transcriptome sequencing, benefit from molecular level to the *A. rubrum* leaf color change of a variety of metabolic and information processing ways were analyzed. In this study, 43.21% of the annotated unigenes were longer than 1000 bp, and 32.10% were between 300 and 1000 bp. In the biological process category, the subcategory most enriched with DEGs was metabolic process, indicating that girdling affected biological functions in red maple. Transcriptome analysis of leaves from girdled branches revealed up-regulation of c45897.graph_c0, which encodes a transcription factor in the Pkinase gene family that is involved in the regulation of leaf color change. In rice, *OSEDR1* encoding a protein in the Pkinase family was found to be related to a spotted-leaf phenotype [43]. In this study, c108619.graph_c0, also encoding a member of the Pkinase gene family, was identified as one of the factors involved in the regulation of anthocyanin accumulation during leaf color change in red maple.

## Conclusions

The growth and photosynthesis of red maple were significantly inhibited by girdling, but protective mechanisms were activated to maintain normal metabolism in girdled branches. The results of transcriptomic and metabolomic analyses revealed significant changes in the metabolic pathways, biosynthesis of secondary metabolites, and carbon metabolisms in red maple leaves after girdling. Girdling impaired the sieve tubes in the phloem, resulting in the blocking of downward transport of assimilates. This resulted in the accumulation of carbohydrates in the upper part of girdled branches, which provided the material basis for the formation of anthocyanins in leaves. At the same time, photosynthesis was weakened by girdling, while pigments accumulated above the girdling site and the red coloration of leaves was particularly prominent. The GO and KEGG annotation and enrichment analyses showed that the differentially expressed genes in leaves between girdled and non-girdled branches were involved in biological processes such as primary metabolism, secondary metabolism, and carbon metabolism. Six anthocyanin-related transcription factors were up-regulated in leaves above the girdling site, and these transcription factors are known to regulate phenylpropanoid biosynthesis, anthocyanin biosynthesis, and flavonoid biosynthesis. These six transcription factors were found to be highly expressed in red or mosaic leaves, providing further evidence for their role in regulating color change in red maple leaves. These results suggest that leaf reddening is a complex environmental adaptation strategy to maintain normal metabolism in response to environmental changes. Overall, this study provides deeper and more reliable insights into the coevolution of red maple leaves in response to environmental change through a combination of phenotypic, physiological, biochemical, transcriptome, and metabolomic analyses.

## Materials and methods

### Plant materials and experimental design

This study involved materials were *A. rubrum* container seedlings introduced from the United States in March 2011, and planted in the south Campus of Southwest University (106°25’45″E, 29°49’ 18″N), with row spacing of 1 m×1 m. All seedlings are managed uniformly. The research area belongs to the subtropical monsoon humid climate, with the annual average temperature of 16 ~ 18℃ and the annual average precipitation of 1000 ~ 1350 mm, most of which is concentrated in May to September. The annual average relative humidity is 70%~80%. The annual sunshine duration is 1000 ~ 1400 h, and the sunshine percentage is only 25%~35%, with less sunshine in winter and spring. Thirty 3-year-old open-grown *A. rubrum* L. (var. *Brandywine*) trees at the Forest Experimental Field at Southwest University, Chongqing, China, were randomly selected, and girdled on one of their main branches with glass fiber (width about 2 cm) in March 2013. Five girdled branches with leaf colors reaching peak red intensity in the 3 days from 6th to 8th November 2016 were selected for subsequent analyses. On each selected tree (height 160–190 cm, trunk diameter at ground height about 3–4 cm), the girdled branch was divided into upper stem and below stem by string, and a nearby stem of similar growth conditions was measured as control. All measurements were conducted at harvest. The foliar coloration (parameters L*, a*, b*) was measured by Photoshop CS3 program (Adobe Systems, CA, USA), which were photographed with a D7000 digital camera (Nikon Corporation, Tokyo, Japan) [44].

### Analyses of photosynthetic pigments

The photosynthetic pigments (chlorophyll *a*, chlorophyll *b*, chlorophyll(*a* + *b*), and carotenoid) contents were measured in foliar methanol extracts as described [45]. The chlorophyll content of leaf was extracted with 95% (v/v) methanol. The extract was assessed at three wave length (λ = 665 nm, λ = 649 nm, λ = 470 nm) (uv-spectrophotometer, T6 New Century, Purkinje General, Beijing, China), which was filtrated and diluted with deionized water to 25mL. Chlorophyll a, chlorophyll b and carotenoid were based on the equations:

$${\mathrm C}_{\mathrm a}=13.95{\mathrm A}_{665}-6.88{\mathrm A}_{649},\;{\mathrm C}_{\mathrm b}=24.96{\mathrm A}_{649}-7.32{\mathrm A}_{665}$$

$$Cx\cdot c=\frac{1000A470-2.05Ca-114.8Cb}{245}$$

Measurement of anthocyanin and flavonoid were following the method of Zhang [46]. The anthocyanin was extracted by 1% (v/v) acidic alcohol under 32℃ water bath for four hours, then separated at 5000r/min for ten minutes. Flavonoid was measured at 367 nm (DU series 730 spectrophotometers, Beckman Coulter, USA), which was extracted with 70% methanol by ultrasound and create complexes with 10% aluminium nitrate under mixed liquor (60% alcohol, 5% sodium nitrate and 4% sodium hydroxide). Soluble sugar was extracted with deionized water in boiling water bath for twice (three minutes each time), and measured at 630 nm (uv-spectrophotometer(New Century T6), Shanghai child analysis instrument co., LTD, Beijing, China) [47].

### Sample preparation and extraction

The freeze-dried leaf was crushed using a mixer mill (MM 400, Retsch) with a zirconia bead for 1.5 min at 30 Hz. 100 mg powder was weighted and extracted overnight at 4℃ with 0.6 ml 70% aqueous methanol. Following centrifugation at 10, 000 g for 10 min, the extracts were absorbed (CNWBOND Carbon-GCB SPE Cartridge, 250 mg, 3ml; ANPEL, Shanghai, China, www.anpel.com.cn/cnw) and filtrated (SCAA-104, 0.22 μm pore size; ANPEL, Shanghai, China, http://www.anpel.com.cn/) before UPLC-MS/MS analysis.

### UPLC conditions

The sample extracts were analyzed using an UPLC-ESI-MS/MS system (UPLC, Shim-pack UFLC SHIMADZU CBM30A system, www.shimadzu.com.cn/; MS, Applied Biosystems 4500 Q TRAP, www.appliedbiosystems.com.cn/). The analytical conditions were as follows, UPLC: column, Agilent SB-C18 (1.8 μm, 2.1 mm*100 mm). The mobile phase consiste of solvent A, pure water with 0.1% formic acid, and solvent B, acetonitrile. Sample measurements were performed with a gradient program that employed the starting conditions of 95% A, 5% B. Within 9 min, a linear gradient to 5% A, 95% B was programmed, and a composition of 5% A, 95% B was kept for 1 min. Subsequently, a composition of 95% A,5.0% B was adjusted within 1.10 min and kept for 2.9 min. The column oven was set to 40 °C; The injection volume was 4 µl. The effluent was alternatively connected to an ESI-triple quadrupole-linear ion trap (QTRAP)-M.

#### ESI-Q TRAP-MS/MS

LIT and triple quadrupole (QQQ) scans were acquired on a triple quadrupole-linear ion trap mass spectrometer (Q TRAP), API 4500 Q TRAP UPLC/MS/MS System, equipped with an ESI Turbo Ion-Spray interface, operating in positive and negative ion mode and controlled by Analyst 1.6.3 software (AB Sciex). The ESI source operation parameters were as follows: ion source, turbo spray; source temperature 550℃; ion spray voltage (IS) 5500 V (positive ion mode)/-4500 V (negative ion mode); ion source gas I (GSI), gas II(GSII), curtain gas (CUR) were set at 50, 60, and 30.0 psi, respectively; the collision gas(CAD) was high. Instrument tuning and mass calibration were performed with 10 and 100 µmol/L polypropylene glycol solutions in QQQ and LIT modes, respectively. QQQ scans were acquired as MRM experiments with collision gas (nitrogen) set to 5 psi. DP and CE for individual MRM transitions was done with further DP and CE optimization. A specific set of MRM transitions were monitored for each period according to the metabolites eluted within this period.

#### RNAseq

Quantitative samples were homogenized with TRIzol reagent (Invitrogen, USA). After centrifugation and shaking, sodium acetate solution, water-saturated solution and chloroform/isoamyl alcohol were added, centrifuging and extracting at low temperature for 15 min. The total RNA solution was obtained by rinsing with 75% ethanol, collecting the precipitate and adding DEPC treated water.

#### Transcriptomics

The mRNA separated with magnetic bead method was interrupted by TruseqTM RNA Sample Prep Kit (Illumina, USA) reagent ions. After synthesis, complementing, and amplification of the double-stranded cDNA, the target bands were recovered using 2% agarose gum (Certified Low Range Ultra Agarose, BIO-RAD, USA). The obtained target bands were sequenced quantitatively with Illumina Hiseq sequencing platform (2 × 150 bp) to obtain clusters. The sequencing data were analyzed by DAVID online analysis tool for GO, KEGG, NR, Swiss-Prot and other functional cluster analysis.

#### Metabolomics

To obtain target gene groups and main differential metabolites regulating leaf discoloration, fresh samples were selected for low temperature extraction (at -20℃ for 30 min and then melted (4℃), the frozen methanol/water (1:1) palliative solution was added, the Tissue Lyser was ground for 5 min and centrifuged at low temperature). The extract was analyzed by liquid chromatograph (Liquid phase parameters: ACQUITY UPLC BEH C18 Column (100 mm×2.1 mm, 1.7 μm, Waters, UK), column temperature: 50 ℃, flow rate: 0.4 mL•min^− 1^, 2777 C UPLC System, Waters, UK). Small molecules eluted from the column were collected in MSE mode with a High resolution Tandem Mass Spectrometer (Xevo G2-XS QTOF, Waters, UK). After format transformation, noise filtering, peak matching and peak extraction, the obtained original data was analysed with partial least squares discriminant method.

### Statistics

All charts were drawn with mean value and standard error of parameters by Origin Pro 8.0. Tables were made by Microsoft Office Excel 2007 (Microsoft, Redmond, WA). Significance of differences in the measured parameters among upper stem, lower stem and control branch was tested by Duncan(D) test of one-way ANOVA analysis. All statistical analyses were performed with SPSS 17.0 statistics software (SPSS Inc., Chicago, IL).

#### Hierarchical cluster analysis and Pearson correlation coefficients

The HCA (hierarchical cluster analysis) results of samples and metabolites were presented as heatmaps with dendrograms, while pearson correlation coefficients (PCC) between samples were caculated by the cor function in R and presented as only heatmaps. Both HCA and PCC were carried out by R package pheatmap. For HCA, normalized signal intensities of metabolites (unit variance scaling) are visualized as a color spectrum.

#### Differential metabolites selected

Significantly regulated metabolites between groups were determined by VIP ≥ 1 and absolute Log2FC (fold change) ≥ 1. VIP values were extracted from OPLS-DA result, which also contain score plots and permutation plots, was generated using R package MetaboAnalyst R. The data was log transform (log2) and mean centering before OPLS-DA. In order to avoid overfitting, a permutation test (200 permutations) was performed.

#### Differential expressed genes selected

Differential gene expression gene sets were obtained by analysis of differences between sample groups, which The screening conditions for differential genes were Log2FC (fold change) ≥ 1 and FDR < 0.05. After the difference analysis, the Benjamini-Hochberg method was used to conduct multiple hypothesis testing correction for hypothesis testing probability (P value).

#### KEGG annotation and enrichment analysis

Identified metabolites were annotated using KEGG Compound database (http://www.kegg.jp/kegg/compound/), annotated metabolites were then mapped to KEGG Pathway database (http://www.kegg.jp/kegg/pathway.html). Pathways with significantly regulated metabolites mapped to were then fed into MSEA (metabolite sets enrichment analysis), their significance was determined by hypergeometric test’s p-values.

## Data Availability

The datasets generated and/or analysed during the current study are available in the NCBI repository, https://dataview.ncbi.nlm.nih.gov/object/PRJNA855164?reviewer=veskfcgf2qojnk5lat3nbe9481. The data used to support the findings of this study are available from the corresponding author upon request.

## Abbreviations

CB: Control branch
KEGG: Kyoto Encyclopedia of Genes and Genomes
KOG: Eukaryotic Orthologous Groups
GO: Gene Ontology
US: Upper Stem
LS: Lower stem
PCC: Pearson Correlation Coefficients

## References

[1] Rachel A, Peter R (2015). Early Autumn Senescence in Red Maple (*Acer rubrum* L.) Is Associated with High Leaf Anthocyanin Content. Plants.

[2] Yun Z, Yves B, Xiu-Hai Z, et al. Stand history is more important than climate in controlling red maple (*Acer rubrum* L.) growth at its northern distribution limit in western Quebec, Canada. J Plant Ecol. 2015:8(4):368.

[3] Jian Z. The overview of technique research on *Acer rubrum* of American colorful-leaf trees. J Guangxi Agriculture. 2009.

[4] Ren J, Zeng-Cheng D, F Tang, et al. A New Acer rubrum Cultivar ‘Jinmaihong’. Acta Horticulturae Sinica, 2013.

[5] Ferreyra MLF, Rius SP, Casati P (2012). Flavonoids: biosynthesis, biological functions, and biotechnological applications. Front. Plant Sci.

[6] Luke J, Cooney H, Martin S, Barry A, Logan, Bart C (2015). Gould. Functional significance of anthocyanins in peduncles of *Sambucus nigra*. Environ Experiment Botany.

[7] Franck N, Vaast P, Genard M, Dauzat J (2006). Soluble sugars mediate sink feedback down-regulation of leaf photosynthesis in fild-grown *Coffea arabica*. Tree Physiol.

[8] Shinichi A, Ryan MG (2015). Carbohydrate regulation of photosynthesis and respiration from branch girdling in four species of wet tropical rain forest trees. Tree Physiol.

[9] Tao W, An Y, Zhao C, et al. Regulation Effects of Crataegus pinnatifida Leaf on Glucose and Lipids Metabolism. J Agricult Food Chem. 2011;59(9):4987–4994. 10.1021/jf1049062 21425878

[10] Cutanda-Perez MC, Ageorges A, Gomez C (2009). Ectopic expression of VlmybA1 in grapevine activates a narrow set of genes involved in anthocyanin synthesis and transport. Plant Mol Biol.

[11] Weiss D (2010). Regulation of flower pigmentation and growth: Multiple signaling pathways control anthocyanin synthesis in expanding petals. Physiologia Plantarum.

[12] Zf A, Hs A, Hui JA (2020). Systematic Identification of the Light-quality Responding Anthocyanin Synthesis-related Transcripts in Petunia Petals - ScienceDirect. Horticult Plant J.

[13] Takos A M, Felix W,Jaffé, Jacob S R, et al. Light-induced expression of a MYB gene regulates anthocyanin biosynthesis in red apples. Plant Physiol. 2006:142;(3):12–32. 10.1104/pp.106.088104 PMC1630764 17012405

[14] Murakami PF, Schaberg PG, Shane JB (2008). Stem girdling manipulates leaf sugar concentrations and anthocyanin expression in sugar maple trees during autumn. Tree Physiol.

[15] Fleschhut J, Kratzer F, Rechkemmer G (2006). Stability and biotransformation of various dietary anthocyanins in vitro. Eur J Nutri.

[16] Konishi T (2004). Evidence that sucrose loaded into the phloem of a poplar leaf is used directly by sucrose synthase associated with various beta-glucan synthases in the stem. Plant Physiol.

[17] Urban Laurent Mathieu, Lechaudel, Lu Ping (2004). Effect of fruit load and girdling on leaf photosynthesis in *Mangifera indica* L. J Experiment Botany.

[18] Regier N, Streb S, Zeeman SC (2010). Seasonal changes in starch and sugar content of poplar (*Populus deltoides* × nigra cv. Dorskamp) and the impact of stem girdling on carbohydrate allocation to roots. Tree Physiol.

[19] Li P, Ponnala L, Gandotra N (2010). The developmental dynamics of the maize leaf transcriptome. Nat Genet.

[20] Guo Y, Cai Z, Gan S (2010). Transcriptome of Arabidopsis leaf senescence. Plant Cell Environ.

[21] Krol M, Gray GR, Hurry VM, Öquist G, Malek L (1995). Low-temperature stress and photoperiod affect an increased tolerance to photoinhibition in *Pinus banksiana* seedlings. Can J Botany.

[22] Luke J, Cooney H, Martin S, Barry A, Logan (2015). Functional significance of anthocyanins in peduncles of *Sambucus nigra*. Environment Experiment Botany.

[23] Sperdouli I, Moustakas M (2012). Interaction of proline, sugars, and anthocyanins during photosynthetic acclimation of Arabidopsis thaliana to drought stress. J Plant Physiol.

[24] Cirillo V, D’Amelia V, Esposito M, Amitrano C, Maggio A (2021). Anthocyanins Are Key Regulators of Drought Stress Tolerance in Tobacco. Biology.

[25] Dale MP, Causton DR (1992). The Ecophysiology of Veronica Chamaedrys, V. Montana and V. Officinalis. IV. Effects of Shading on Nutrient Allocations–A Field Experiment. J Ecol.

[26] Johansson T (2008). Seasonal changes in contents of root starch and soluble carbohydrates in 4–6-year old *Betula pubescens* and *Popuius tremula*. Scand J Forest Res.

[27] Bollmark L, Lisa S, Tom E (1999). Seasonal dynamics and effects of nitrogen supply rate on nitrogen and carbohydrate reserves in cutting-derived *Salix viminalis* plants. Revue Canadienne De Recherche Forestière.

[28] Dunn JP, Lorio PL (1992). Effects of bark girdling on carbohydrate supply and resistance of loblolly pine to southern pine beetle (*Dendroctonus frontalis* Zimm.) attack. Forest Ecol Manag.

[29] Jordan MO (1996). Mobilizable carbon reserves in young peach trees as evidenced by trunk girdling experiments. J Experiment Botany.

[30] Ishii H, Hama Da Y, Utsugi H (2012). Variation in light-intercepting area and photosynthetic rate of sun and shade shoots of two *Picea* species in relation to the angle of incoming light. Tree Physiol.

[31] Kyparissis A, Grammatikopoulos G, Manetas Y (2007). Leaf morphological and physiological adjustments to the spectrally selective shade imposed by anthocyanins in *Prunus cerasifera*. Tree Physiol..

[32] Manetas Y, Petropoulou Y, Psaras GK, Drinia A (2003). Exposed red (anthocyanic) leaves of *Quercus coccifera* display shade characteristics. Funct Plant Biol.

[33] Kasuga J, Arakawa K, Fujikawa S (2007). High accumulation of soluble sugars in deep supercooling Japanese white birch xylem parenchyma cells. New Phytolog.

[34] Gould KS, Vogelmann TC, Han T, Clearwater MJ (2002). Profiles of photosynthesis within red and green leaves of *Quintinia serrata*. Physiologia Plantarum.

[35] Lev-Yadun S, Yamazaki K, Holopainen KJ, Sinkkonen A. Spring versus autumn leaf colours: Evidence for different selective agents and evolution in various species and floras. FLORA -JENA-. 2012:207:6:80–85.

[36] Lev-Yadun S. Spring Versus Autumn or Young Versus Old Leaf Colors: Evidence for Different Selective Agents and Evolution in Various Species and Floras. Defensive (anti-herbivory) Coloration in Land Plants. 2016:259–266.

[37] Hughes NM (2011). Winter leaf reddening in ‘evergreen’ species. New Phytolog.

[38] Becker C, Klaering HP, Kroh LW, Krumbein A (2014). Cool-cultivated red leaf lettuce accumulates cyanidin-3-O-(6’’-O-malonyl)-glucoside and caffeoylmalic acid. Food Chem.

[39] Kraj W (2017). Stem Girdling Affects the Carbon/Nitrogen Imbalance and Oxidative Stress, and Induces Leaf Senescence in Phenological Forms of Beech (Fagus Sylvatica). Acta Biologica Cracoviensia S Botanica.

[40] Zhang HW, Kuan-Xin HE, Cheng XQ, Liu LW (2015). Effects of Irrigation and Nitrogen on Carbon Metabolism, Growth and Quality of Flue-cured Tobacco in Dry-land Purplish Soil. Acta Agriculturae Boreali-Sinica..

[41] Mishra V, Srivastava G, Prasad SM, Abraham G (2008). Growth, photosynthetic pigments and photosynthetic activity during seedling stage of cowpea (*Vigna unguiculata*) in response to UV-B and dimethoate. Pesticide Biochem Physiol.

[42] Han XB, Xu R, Duan PG, Yu HY, Li YH, Li YH. Genetic analysis and identification of candidate genes for two spotted-leaf mutants (spl101 and spl102) in rice. Hereditas. 2017:39(4): 346–353. 10.16288/j.yczz.16-416 28420613

[43] Causton M (1992). Use of the chlorophyll a/b ratio as a bioassay for the light environment of a plant. Funct Ecol.

[44] Yan Y, Liu Y, Liu Y, Li L, Li J, Zhou W (2018). Long-term banding modifies the changes to foliar coloration of *Acer rubrum* L. ‘Brandywine’. Scientia Horticulturae.

[45] Yemm EW, Willis AJ (2010). Stomatal movements and changes of carbohydrate in leaves of chrysanthemum maximum. New Phytologist.

[46] Zhang Y, Hu Z, Chu G, Huang C, Tian S, Zhao Z, Chen Z (2014). Anthocyanin Accumulation and Molecular Analysis of Anthocyanin Biosynthesis-Associated Genes in Eggplant (*Solanum melongena* L.). J Agricult Food Chemi.

[47] Xu F, Cheng SY, Zhu J, Zhang WW, Wang Y (2011). Effects of 5-Aminolevulinic Acid on Chlorophyll, Photosynthesis, Soluble Sugar and Flavonoids of Ginkgo biloba. Notulae Botanicae Horti Agrobotanici Cluj-Napoca..

